# Pitch Syntax Violations Are Linked to Greater Skin Conductance Changes, Relative to Timbral Violations – The Predictive Role of the Reward System in Perspective of Cortico–subcortical Loops

**DOI:** 10.3389/fpsyg.2017.00586

**Published:** 2017-04-18

**Authors:** Edward J. Gorzelańczyk, Piotr Podlipniak, Piotr Walecki, Maciej Karpiński, Emilia Tarnowska

**Affiliations:** ^1^Department of Theoretical Basis of Bio-Medical Sciences and Medical Informatics, Nicolaus Copernicus University Collegium MedicumBydgoszcz, Poland; ^2^Non-Public Health Care Center Sue Ryder HomeBydgoszcz, Poland; ^3^Medseven—Outpatient Addiction TreatmentBydgoszcz, Poland; ^4^Institute of Philosophy, Kazimierz Wielki UniversityBydgoszcz, Poland; ^5^Institute of Musicology, Adam Mickiewicz University in PoznańPoznań, Poland; ^6^Department of Bioinformatics and Telemedicine, Jagiellonian University Collegium MedicumKrakow, Poland; ^7^Institute of Linguistics, Adam Mickiewicz University in PoznańPoznań, Poland; ^8^Institute of Acoustics, Adam Mickiewicz University in PoznańPoznań, Poland

**Keywords:** pitch syntax, prediction, cortico–subcortical loops, skin conductance, timbre

## Abstract

According to contemporary opinion emotional reactions to syntactic violations are due to surprise as a result of the general mechanism of prediction. The classic view is that, the processing of musical syntax can be explained by activity of the cerebral cortex. However, some recent studies have indicated that subcortical brain structures, including those related to the processing of emotions, are also important during the processing of syntax. In order to check whether emotional reactions play a role in the processing of pitch syntax or are only the result of the general mechanism of prediction, the comparison of skin conductance levels reacting to three types of melodies were recorded. In this study, 28 subjects listened to three types of short melodies prepared in Musical Instrument Digital Interface Standard files (MIDI) – tonally correct, tonally violated (with one out-of-key – i.e., of high information content), and tonally correct but with one note played in a different timbre. The BioSemi ActiveTwo with two passive Nihon Kohden electrodes was used. Skin conductance levels were positively correlated with the presented stimuli (timbral changes and tonal violations). Although changes in skin conductance levels were also observed in response to the change in timbre, the reactions to tonal violations were significantly stronger. Therefore, despite the fact that timbral change is at least as equally unexpected as an out-of-key note, the processing of pitch syntax mainly generates increased activation of the sympathetic part of the autonomic nervous system. These results suggest that the cortico–subcortical loops (especially the anterior cingulate – limbic loop) may play an important role in the processing of musical syntax.

## Introduction

Tonal music is a natural, and complex syntactic system (Lerdahl and Jackendoff, 1983) based on implicitly learned norms (Tillmann et al., 2000; Tillmann, 2005). The perception of pitch structure as hierarchically organized discrete units (pitch classes) is an important part of syntactic processing in music (Krumhansl, 1990; Krumhansl and Cuddy, 2010; Lerdahl, 2013). During this process, the recognition of each pitch class in the context of other pitch classes is accompanied by subtle emotional sensations known as tension, uncertainty, stability, completion, and power, etc. which are often referred to as ‘tonal qualia’ (Huron, 2006; Margulis, 2012). According to Huron (Huron, 2006), positive emotions (e.g., the tonal qualia of resolution or completeness often described as pleasure, contentment etc.) are the result of limbic reward for accurate predictions, whereas negative emotions (e.g., the tonal qualia of tension or incompleteness often described as uncomfortable, jarring, anxious etc.) are elicited in case our predictions are inaccurate. This claim is in line with classic Darwinian rules (Darwin, 1859) as emotions actually enable the adaptation of behavior to particular circumstances (Darwin, 1872; Panksepp, 1998). Accordingly animals, including mammals, are able to continuously predict changes in their environment. More accurate prediction implies more probable survival. Therefore, various emotions are elicited depending on the different probabilities of occurring events. Since the probabilities of pitch class occurrences depend on the context of other pitch classes, then various levels of prediction accuracy result in slightly diverse emotional sensations. Because the reward system plays a crucial role in the prediction of perceived stimuli by the means of emotional control (Abler et al., 2006; Juckel et al., 2006), the concept of cortico–subcortical loops may help to provide a basic explanation of these phenomena (Gorzelańczyk, 2011). The main structure of the reward system is the ventral striatum, which is a crucial part of the limbic loop (Abler et al., 2006). The limbic system controls the functions of the autonomic nervous system (Mujica-Parodi et al., 2009; Tavares et al., 2009) and the endocrine system (Herman et al., 2005; Krüger et al., 2015; Tasker et al., 2015). The activity of the latter influences skin conductance (Dawson et al., 2007; Zhang et al., 2016) and other physiological parameters (Wood et al., 2014; Schröder et al., 2015).

**FIGURE 1:**
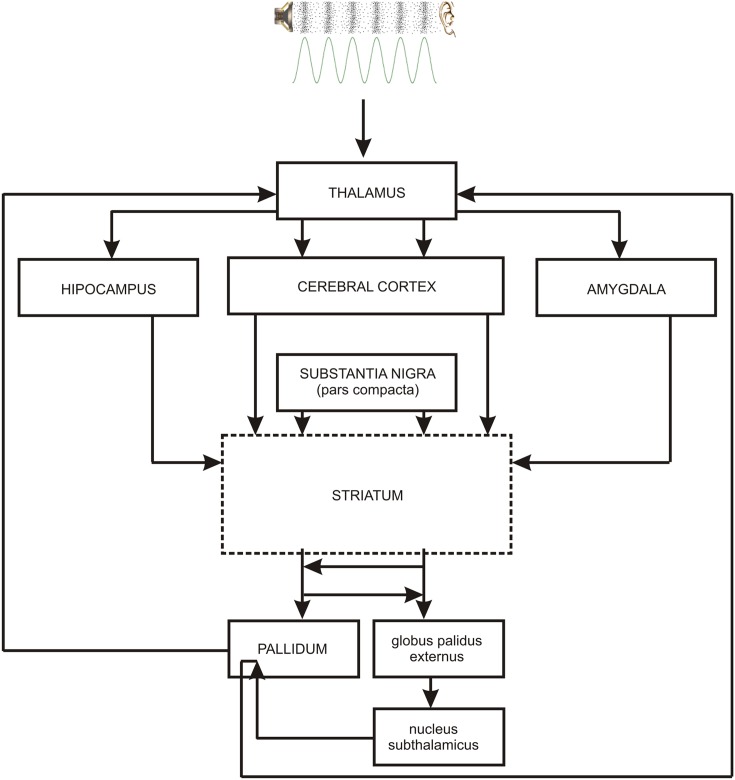
**The main brain structures of cortico–subcortical loops: striatum – cerebral cortex – pallidum.** PALLIDUM = globus palidus internalis + substantia nigra reticulata + ventral part of the globus palidus. Contiguous brain structures (hippocampus, amygdala) are also shown. The broken line indicates the main structure of every loop.

**FIGURE 2:**
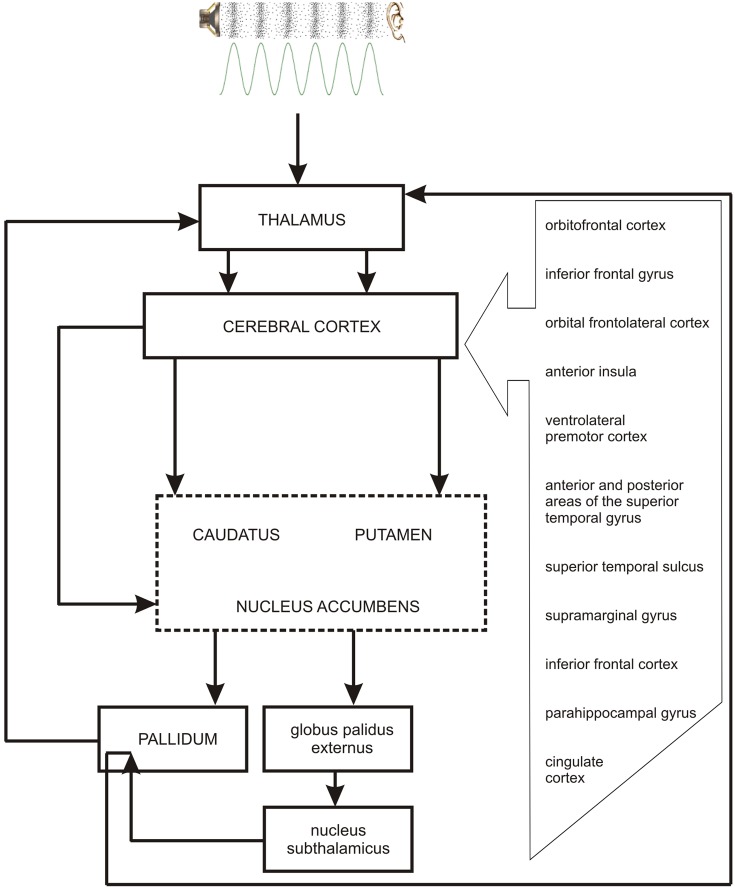
**The link between the perception of pitch structure (all the cortical structures depicted in the figure and striatum), emotions (nucleus accumbens – the central structure of the limbic loop, amygdala), prediction (cingulate cortex, striatum), and the reward system (nucleus accumbens, hippocampus, amygdala)**.

**FIGURE 3:**
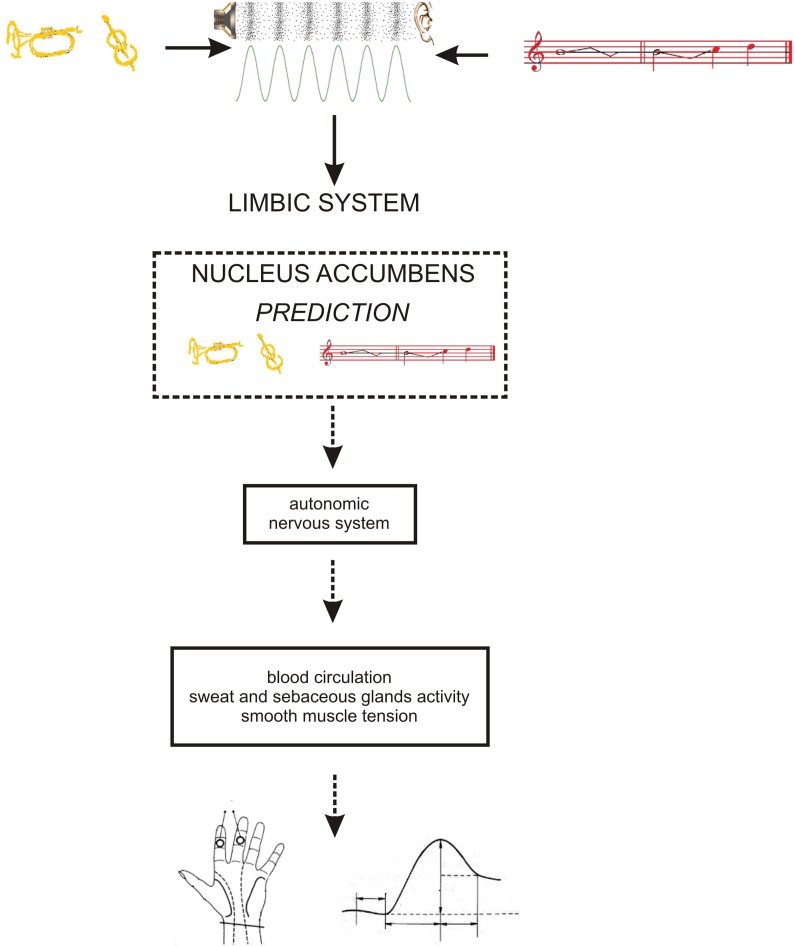
**The functional relationship between: limbic system – autonomic nervous system – skin conductance.** Timbre = yellow; pitch structure = red.

As the occurrence of out-of-key notes in tonal melody is usually very improbable (Pearce and Wiggins, 2006; Pearce et al., 2010; Hansen and Pearce, 2014), the response of the reward system should be stronger than in the case of ‘in-key’ notes. In fact, a number of studies indicate that the perception of a violated tonal structure leads to measurable somatic reactions such as changes of skin conductance (Steinbeis et al., 2006; Koelsch et al., 2008b). There are also studies that show the modulation in the activity of the amygdala (Koelsch et al., 2008a; Mikutta et al., 2015), the orbitofrontal cortex (Tillmann et al., 2003; Koelsch et al., 2005; Mikutta et al., 2015), the inferior frontal gyrus, the orbital frontolateral cortex, the anterior insula, the ventrolateral premotor cortex, the anterior and posterior areas of the superior temporal gyrus, the superior temporal sulcus, the supramarginal gyrus (Koelsch et al., 2005), the inferior frontal cortex (Tillmann et al., 2003, 2006), the parahippocampal gyrus and the cingulate cortex (Omigie et al., 2015), depending on the expectedness of musical pitch structure. Information processed by various cortico–subcortical loops partly overlap in the striatum where it is exchanged between particular circuits (**Figure 1**). The certain parts of the striatum (the caudatus, the putamen, the nucleus accumbens) are connected to specific areas of the cerebral cortex. Sensorimotor cortices are connected with the putamen, association cortices are connected to the caudatus, and limbic cortices and the amygdala are connected with the nucleus accumbens (**Figure 2**). The striatum, especially the ventral striatum (nucleus accumbens) (Kruse et al., 2016), as well as the amygdala, and the orbitofrontal cortex (Tabbert et al., 2006) are strictly physiologically connected to the autonomic nervous system which controls the vegetative functions of the body (the cardiovascular system, the endocrine system, and the digestive system) including blood circulation in the skin, the activity of sweat and sebaceous glands, and smooth muscle tension in the skin (**Figure 3**). These physiological responses can cause changes in the electrical conductance of the skin (Johnson and Corah, 1963; Grove, 1992; Alonso et al., 1996; Klucken et al., 2009; Benedek and Kaernbach, 2010; Boucsein, 2014). This can explain why the perception of violated tonal structure can lead to measurable somatic reactions such as changes in skin conductance (Steinbeis et al., 2006; Koelsch et al., 2008b). Therefore, it is not surprising that the modulation of the activity in the amygdala (closely linked to the nucleus accumbens and the limbic system) (Koelsch et al., 2008a; Mikutta et al., 2015) changes value of skin conductance. The latero-orbito-frontal and limbic loops are particularly important in the control of executive functions (Alexander et al., 1986; Royall et al., 2002; Haber, 2003), which may explain why the activation of the orbitofrontal cortex (Tillmann et al., 2003; Koelsch et al., 2005; Mikutta et al., 2015) can change skin conductance. The fact that the anterior cingulate loop is responsible for correcting behavior following a mistake (Peterson et al., 1999) and that the parahippocampal gyrus is strictly connected to the limbic system, are consistent with observations that the activation of the parahippocampal gyrus and the cingulate cortex (Omigie et al., 2015) are related to the changing expectedness of the components of musical pitch structure.

Even a very simple melody is a complex stimulus, composed of sounds characterized by not only the fundamental frequency (*F*_0_), which mainly influences the sensation of pitch (Stainsby and Cross, 2008), but also by other acoustic parameters. Our predictions also include some of these parameters. For example, spectral centroid, attack time, and spectral irregularity influence the sensation of timbre in music (McAdams and Giordano, 2008). Although it may seem that timbre in music is not perceptively organized in a hierarchical way, the specific characteristics of spectral (e.g., spectral centroid) and temporal (e.g., attack time) cues allow for the discrimination between different categories of timbres, e.g., between the timbre of the flute and the piano. These categories, similar to pitch, occur in music with different probabilities. For example, the probability that a melody played in a particular timbre will suddenly change (e.g., from piano to flute) seems to be even lower than the occurrence of an out-of-key note. After all, the vast majority of musical tunes which are experienced by listeners living in Western society are sung or played by the same singer or the same musical instrument. Of course, some spectral changes can occur even when a melody is sung or played by one musical source. However, from a cognitive point of view these spectral deviations do not break the perceptual congruity of a percept as belonging to one timbral category. If a human reaction to sound probabilities depends solely on the general mechanism of prediction, then timbral changes should cause a reaction of the autonomic nervous system at least as strong as that to an out-of-key note. Apart from that, in contrast to a small change in pitch (out-of-key note), changes in timbre cause a violation in the auditory stream (Wessel, 1979; Gregory, 1994; Huron, 2016) which should also elicit a stronger reaction of the autonomic nervous system than in the case when the auditory stream is preserved when the whole melody is played in the same timbre. We hypothesize that pitch structure in music is processed by a domain-specific circuit which differs functionally from that responsible for the predictions of timbre. Although both circuits implement the predictive mechanisms of cortico–subcortical loops (Gorzelańczyk, 2011), only the activity of the pitch class prediction circuit results in syntactical organization of perceived sounds in music which allows for the recognition of a recursive relationship (Woolhouse et al., 2016). According to behavioral observations, emotional reaction related to prediction is a specific part of the recognition of pitch hierarchy (Huron, 2006). This specificity should be possible to recognize by measuring the somatic markers of autonomic nervous system activity. In other words, the reaction of the autonomic nervous system to an out-of-key note should be different from the reaction to an in-key note and to a change of timbre.

## Materials and Methods

### Stimuli

**FIGURE 4:**
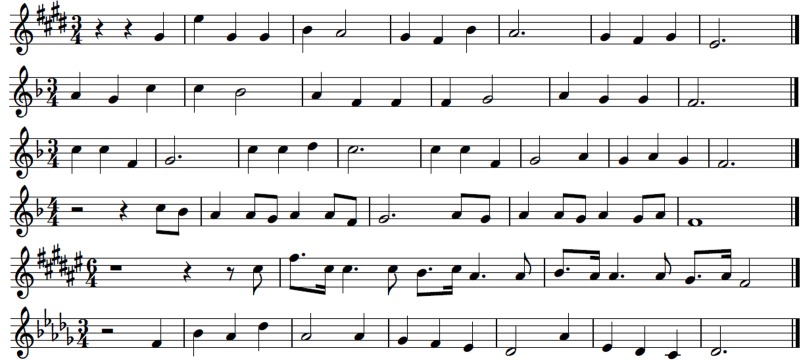
**Tonally correct melodies**.

**FIGURE 5:**
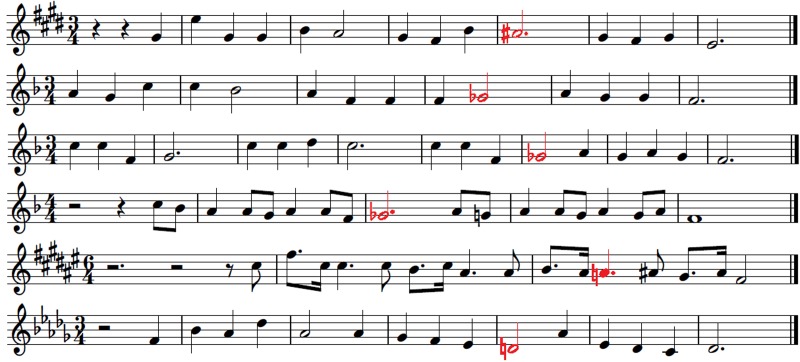
**Tonally violated melodies.** Out-of-key notes are red.

**FIGURE 6:**
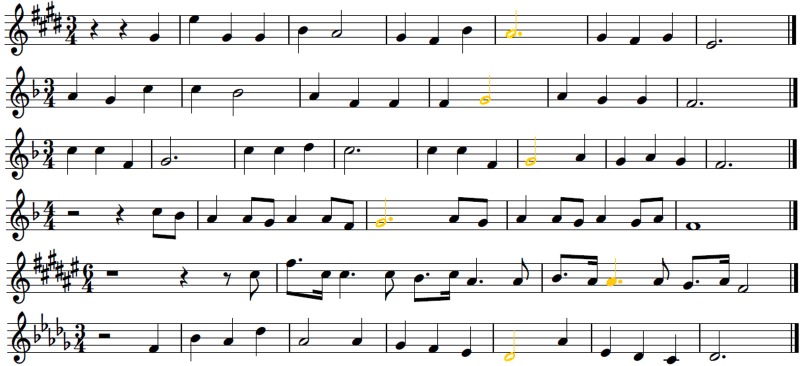
**Tonally correct melodies with one note played in a different timbre (flute instead of piano timbre).** The notes played in a different timbre are in yellow.

Six simple tonal melodies were prepared as MIDI files without any dynamics and tempo changes in order to avoid additional expressive content. Each melody lasted for 6–12 s. The key signatures of all melodies were chosen randomly in order to avoid the exposure effect as a result of the latent memory of pitch. From the MIDI files of these melodies six additional MIDI files were generated so that one note in each basic melody was changed into an out-of-key note. In order to avoid possible interpretation of the results as just a reaction to interval change or scale degree, different intervals leading to out-of-key notes and different scale degrees of changed pitches were chosen. Because musical rhythm and meter is also processed in the brain by the means of predictive coding (Vuust and Witek, 2014), the locations of out-of-key notes were placed randomly in the bars in order to exclude the possible influence of the same metrical stress (or lack of stress) on skin conductance reactions. Apart from this, six further MIDI files were prepared. This time the timbre of one note in each basic melody was changed instead of the fundamental frequency. The notes of the basic melodies in which the timbre was changed were exactly the same notes to those previously changed into out-of-key. Each change was made after 8 to 12 notes of each melody. As a result there were three versions of six melodies: tonally correct (**Figure 4**), tonally violated – with an out-of-key note (**Figure 5**), and tonally correct but with one note played in a different timbre (**Figure 6**).

### Method

Twenty eight musically untrained (people without any formal musical education and who do not play any instrument) medical students (18 women, 10 men; age: mean = 20.21; *SD* = 1.55) were studied. The research was conducted on people who voluntarily participated in the study. Prior to testing the subjects were informed that some melodies had been modified by replacing one note with another note, an out-of-key note, and some others by changing the timbre of one note. The subjects were then asked to listen for and focus their attention on tonal violation in the stimuli in order to concentrate the attention of the subjects on the stimuli. Before each session, the subjects listened to one example of each type of melody so that they understood what the task would involve. Skin conductance was measured with the ActiveTwo (Biosemi^®^) biopotential measurement system and two passive Nihon Kohden electrodes were placed on the medial phalanges of the index and middle finger of the subject’s non-dominant hand. The current used was 1 μA at 16 Hz, synchronized with a sampling rate of 8.192 kHz. The resolution of measurements was 1 nanoSiemens [*nS*]. The duration of each experimental session (for one person) was ca. 30 min. Individual tests were performed in the same location for all participants. After attaching the electrodes, each participant remained in a quiet and dark place for 15 min. This was for the purpose of calming any emotional arousal and, simultaneously, as an adaptation period, allowing for the equilibration of hydration and sodium at the interface between the skin and the electrode gel. The experimental procedure was based on the presentation of one MIDI file composed of 18 randomly ordered melodies separated by 1 s pauses. Each melody belonged to one of the following groups: tonally correct, tonally violated, and tonally correct but with one note played in a different timbre. Electrodermal activity was continuously measured during the entire time of listening to each melody. For each melody, the difference between the initial value and the maximum value of conductivity was calculated. This study was carried out in accordance with the recommendations of the Bioethics Committee of the Nicolaus Copernicus University in Torun at Collegium Medicum in Bydgoszcz No. KB 416/2008 on 17.09.2008, with written informed consent from all subjects in accordance with the Declaration of Helsinki.

## Results

**FIGURE 7:**
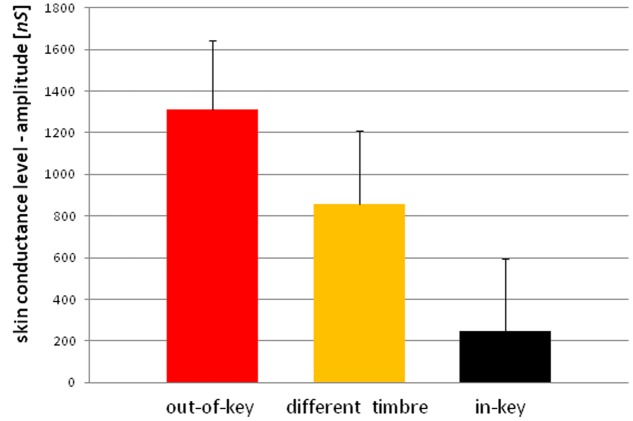
**Skin conductance reactions to tonal violation, timbre change, and in-key notes**.

**FIGURE 8:**
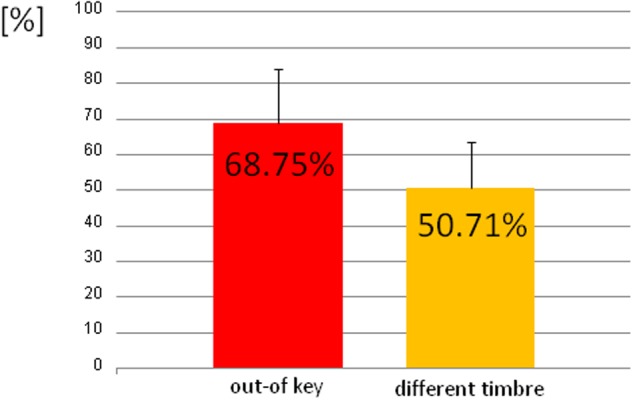
**The percentage of the trials that the participant responded to out-of-key notes and to notes played in a different timbre**.

We observed three types of skin-galvanic activity (**Figure 7**). First was the activity associated with the perception of in-key notes. This activity had a low maximum amplitude (mean = 247.26 [*nS*]; SD = 53.44 [*nS*]). The second reaction was associated with the notes played in a different timbre, the maximum amplitude (mean = 855.32 [*nS*]; *SD* = 144.79 [*nS*]). The third response, associated with the tonally violated notes, was characterized by a high maximum amplitude (mean = 1311.57 [*nS*]; *SD* = 231.12 [*nS*]). Importantly, the subjects differed in the frequency of responses to changed notes. In other words, they did not respond to all out-of-key notes and notes played in a different timbre equally often. Additionally, we observed that the reactions to out-of-key notes were more frequent (68.75% of responses) than to notes played in a different timbre (50.71% of responses) (**Figure 8**).

Because the data did not have a normal distribution (Shapiro–Wilk *W* = 0.90410, *p* = 0.01429) we used the ‘Wilcoxon matched-pairs signed-ranks test.’ The ‘Wilcoxon matched-pairs signed-ranks test’ indicates a statistically significant (*p* < 0.05) difference between the mean maximum amplitude of reactions after in-key notes and the mean maximum amplitude of reactions after out-of-key notes and the notes played in a different timbre. A statistically significant (*t*-test for dependent means, *p* < 0.05) difference was also found between reactions after out-of-key notes and notes played in a different timbre as well as between the frequency of reactions to out-of-key notes and to notes played in a different timbre.

## Discussion

It has been observed that the mean skin conductance value in response to tonal violations is significantly higher in musical stimuli than to a change in timbre. The higher percentage of responses to tonal violations in comparison to responses to the changes in timbre has also been observed. Interestingly, the electro-dermal responses to tonal violations are more frequent and have a higher value in contrast to the electro-dermal response to timbral changes, although listeners reported after the study that they had been more conscious of the timbral changes in comparison to tonal violations. This may suggest that the recognition strategy for tonal violations is different from the one employed for the timbral change recognition despite the fact that their attention was guided toward the tonal changes (instructions). The observed difference also suggests that the biological significance of tonal violations is higher in comparison to the recognition of the timbral change in music, which suggests a potential biological adaptive value of pitch structure (Podlipniak, 2016). The fact that timbre as a source cue is more evolutionarily salient than key or tempo of melody (Schellenberg and Habashi, 2015) indicates that its change should cause a greater physiological reaction than a change in pitch. Our results show, however, that the change of pitch class which is unexpected in terms of pitch structure leads to a greater physiological response. This result suggests that pitch structure may be evolutionarily important too. Whilst listening to a melody, information about the congruency of the listened tune with schematic expectations seems to become more important than the cues of the sound source. Such an effect supports the claim that pitch structure is a part of the species-specific form of vocalization rather than just a culturally changeable tradition of composing sounds on the basis of pitch. In fact, the perception of species-specific forms of vocalization by mammals (Gouzoules and Gouzoules, 2000; Juslin and Scherer, 2008) and birds (Rothenberg et al., 2014) usually contains an emotional/motivational component. Therefore, the obtained results can support the view that pitch structure is an important part of the vocal communication of *Homo sapiens*. Note that the pitch structure here does not refer to the varying perceptive dimension of pitch which is homologous to speech intonation, but to the cognitive dimension of pitch composed of discrete units (pitch classes) which is analogous to digitized sounds in speech, e.g., phonemes (Jackendoff, 2008). An interesting way to compare the biological importance of pitch and timbre would be to measure reactions to timbral and pitch class changes in different environmental conditions. We suspect that different environments (e.g., listening to melodies in the relatively safe environment of a laboratory versus more dangerous circumstances such as in a forest at night) can influence the physiological reactions of the listener. In a less safe environment timbre as a cue of the sound source should be more biologically important than in a laboratory.

Taking into account that skin conductance changes are one of the measureable reactions of the autonomic nervous system tone (mostly the sympathetic part) controlled by the limbic system and the reward system, it can be assumed that the reward system is more susceptible to pitch class changes than to timbral changes. Although, in contrast to timbre, pitch structure is assumed to be a cognitive dimension of music (Barrett and Janata, 2016) it is also possible that the recognition of pitch structure is related to the activity of the subcortical structures which are rarely studied. Therefore, it seems that the most promising way to explore and understand the processing of pitch structure is a model which takes into account the role of cortico–subcortical loops. From physiological perspective, it is possible to infer from the obtained results that the change of timbre is less important for the fluency of tonal music than tonal violation.

Although not all participants responded as equally often to “out-of-key” notes and timbral changes, the skin conductance responses to “out-of-key” notes were significantly more frequent than to the changes of timbre. This may be a result of the instruction delivered before the study. However, if there was a lack of instruction, different subjects’ attentions, depending on possible different listening strategies chosen by the subjects, could influence their reactions. However, according to many observations, unconscious neural activity precedes and influences conscious decisions (Soon et al., 2013). Therefore, it is more probable that the changes of pitch structure were more conspicuous. The fact that people did not react to all instances of change (tonal violation and timbral change) can be explained by either the fact that they did not focus equally well to the presented stimuli or that not all tonal violations and timbral changes, respectively, were equally prominent for them.

The limited range of timbre changes introduced to our stimuli has resulted in some limitations to our conclusions and generalizations. This opens the debate about different possible reactions to other timbre changes. Since different timbres can be interpreted by the nervous system as the labels of different sound sources which may be dangerous or attractive, i.e., emotionally significant, it is probable that changes of notes into such emotionally significant timbres will cause greater changes of skin conductance levels than emotionally neutral timbres. Thus, in further studies, responses to different timbre changes should be measured. Another possibility would be to modify the primary sounds or entire melodies, e.g., by using various playback rates or reverse the sounds in the time domain.

Another serious issue is the cultural influence on perception strategies used by humans in music listening. Because the role of pitch in speech depends on culture as in the case of tonal and non-tonal languages (Ge et al., 2015), it is possible that the importance of pitch syntax and timbre in music can also differ depending on culture. For example the phenomenon of ‘kuchi shōga’ – a Japanese sound symbolism used as an acoustic-iconic mnemonic system (Hughes, 2000) – necessitates elaborate sensitivity to timbral features which can influence the perception of musical timbre by people skilled in this method. In order to address this question adequately, intercultural studies are necessary. A similar question is related to the possible influence of musical training on the sensitivity to timbral features. Some researchers suggest that musical training can cause musicians to process timbre using their brain network which is specialized for music (Marozeau et al., 2013). If this is true then musicians, especially those who are more familiar with contemporary music in which timbral changes are more salient than pitch structure, should be more sensitive to timbral changes. Since the processing of spectral and temporal cues is important for the processing of the phonological aspects of speech (Shannon et al., 1995; Xu et al., 2005) it is possible that the reactions of individuals to the change in timbre may differ and the results would correlate with the acoustic properties of their mother tongue. What is more, non-musicians who use tonal language (e.g., Mandarin) can process acoustical stimuli like musicians (trained and exposed to western music) (Bidelman et al., 2013), so performing such a study on different groups of people (e.g., musicians) should be the next step of this research. In further research, we intend to employ paired musical and speech stimuli in order to directly compare responses to tonal and phonotactic violations.

An interesting question has been whether there is any visual analog for the observed results. For example, syntax in language can cross modalities, which is evident in the case of sign language. Although certain scholars claim that music can also be cross-modal, e.g., as an expression of body movements (Lewis, 2013), in our opinion it is only possible in restricted elements of music. While rhythm can be expressed in dance by the means of movements there is nothing resembling the experience of pitch syntax in other domains of human perception. In other words, tonal relations seem to be unique and specific only to the auditory modality. However, it would be interesting to investigate human physiological reactions to syntactic violations in speech, sign language and music.

## Author Contributions

EG: Substantial contributions to the conception and design of the study and interpretation of data for the work; writing the work and revising it critically for neurobiological content. PP: Substantial contributions to the conception and design of the study and interpretation of data for the work; writing the work and revising it critically for musicological and psychological content. PW: Substantial contributions to the acquisition, analysis, and interpretation of data for the work; writing the work and revising it critically for psychological content. MK: Substantial contributions to the acquisition, analysis, and interpretation of data for the work as well as to the conception and design of the study; writing the work and revising it critically for psycholinguistic and psychomusicological content. ET: Substantial contributions to the acquisition, analysis, and interpretation of data for the work; writing the work and revising it critically for acoustic content. All the authors agreed to be accountable for all aspects of the work in ensuring that questions related to the accuracy or integrity of any part of the work are appropriately investigated and resolved. All authors contributed to the final approval of the version to be submitted.

## Conflict of Interest Statement

The authors declare that the research was conducted in the absence of any commercial or financial relationships that could be construed as a potential conflict of interest.
